# Molecular typing of Campylobacter jejuni diarrhoeal isolates in a hospital in Makokou, Gabon by enterobacterial repetitive intergenic consensus PCR

**DOI:** 10.1099/acmi.0.000947.v3

**Published:** 2025-03-14

**Authors:** Ornella Zong Minko, Jean Fabrice Yala, Rolande Mabika Mabika, Franck Mounioko, Léonce Fauster Ondjiangui, Jose-Miguel Armesto Paula, Junior Eymard Ondo-Hang, Rachel Moyen

**Affiliations:** 1 Bacteriology Laboratory, Medical Analysis Research Unit, Interdisciplinary Center of Medical Research of Franceville (CIRMF), BP 769 Franceville, Gabon; 2 Molecular and Cellular Biology Laboratory, Microbiology Team (LABMC), Agrobiology Research Unit, Masuku University of Sciences and Techniques (USTM), BP 067 Franceville, Gabon; 3 Vector Systems Ecology Unit, Interdisciplinary Center of Medical Research of Franceville (CIRMF), BP 769 Franceville, Gabon; 4 Centre Hospitalier Régional Omar BONGO ONDIMBA de Makokou (CHROBOM), Makokou, Gabon; 5 Laboratory of Cellular and Molecular Biology, Sciences and Techniques Faculty, University Marien Ngouabi, BP 69 Brazzaville, Republic of Congo

**Keywords:** *Campylobacter jejuni*, diarrhoea, Gabon, genotyping, Makokou

## Abstract

**Context*****.** Campylobacter jejuni*is responsible for 80% of the cases of human foodborne bacterial enteric infections worldwide. However, limited data on its genetic diversity exist, especially using the enterobacterial repetitive intergenic consensus PCR (ERIC-PCR). The study aimed to determine the genetic diversity of *C. jejuni* strains isolated from infant diarrhoeal faeces at the Omar Bongo Ondimba Regional Health Center of Makokou (CHROBOM), Gabon.

**Materials and methods.** A total of 58 strains of *C. jejuni* from patients with gastroenteritis were used in this study. The ERIC-PCR method was used to characterize genetic diversity. The binomial manual method via the online analysis system (http://insilico.ehu.es/dice_upgma/) was used to establish the dendrogram and calculate the discriminatory power of the Simpson diversity index (*D*).

**Results.** The genotyping of *C. jejuni* isolates by the ERIC-PCR method revealed a discriminatory index *D*=0.8451, dividing the 58 isolates into 10 clusters, with 33 genotypic profiles, including 22 non-repeated profiles and 11 repeated profiles. These results indicate a rather polymorphic diversity of *C. jejuni* in the Makokou region of Gabon.

**Conclusion.** The high discriminatory diversity index obtained in this study demonstrates the polymorphic richness within *C. jejuni* strains as revealed by the ERIC-PCR method.

## Data Summary

The authors confirm that all supporting data, code and protocols have been provided within the article.

## Introduction

Campylobacteriosis is an infection caused by the bacteria of the genus *Campylobacter*, which is observed in many parts of the world, such as South America, Africa, North America, Asia and Europe [1]. Campylobacteriosis is caused by *Campylobacter jejuni* in more than 80% of the cases, while *Campylobacter coli* is responsible for 10% of the cases, and *Campylobacter lari* and *Campylobacter upsaliensis* are less frequently reported [2]. However, the main origins of these infections in humans are attributed to the handling or consumption of contaminated meat, particularly poultry [3]. Contact with pets and livestock, consumption of contaminated water or raw milk and travel to high-prevalence areas are also considered as risk factors for humans [4]. In developing countries, infection from humans occurs through acute bloody or non-bloody diarrhoea, particularly in children, the elderly and the immunocompromised [5].

Samie *et al.* [2] reported a prevalence of 34.8% of children with gastroenteritis due to *C. jejuni* in Tanzania and 40% of children with diarrhoea due to *C. jejuni* in South Africa.

Although, most of the time, a spontaneous resolution of *Campylobacter* infections is observed, antimicrobial treatment is sometimes required in severe cases [6]. Thus, with the emergence of antimicrobial resistance in *Campylobacter* infections due to the wide uncontrolled use of antimicrobials in agriculture and veterinary medicine, and the difficulty of culturing these strains, *Campylobacter* infections are increasingly becoming an important public health challenge [7]. *Campylobacter* infections, however, are ecologically very varied, and epidemiological and molecular studies reveal great diversity and similarities between these bacterial species [1]. The use of molecular typing methods, therefore, remains the best alternative to assess similarities between isolates and detect *Campylobacter* strains [8].

Molecular typing is becoming crucial to a better understanding of the mechanisms of infections by helping us to study the spread, the clonality relationship between bacterial strains and their geographic distribution [9]. In the field of infection control, molecular typing techniques play a critical role in measuring and identifying the source of initial infection in hospital and environmental epidemics [5].

Molecular typing based on PCR and, in particular, repeated sequences, such as enterobacteria repetitive inter-genic consensus (ERIC) sequences, can be used as molecular tools to assess clonal variability or the genetic relatedness of many bacterial isolates [8]. This enterobacterial repetitive intergenic consensus PCR (ERIC-PCR) genetic fingerprint is one of the fastest molecular typing techniques used for differentiating all strains of Gram-negative bacteria responsible for nosocomial infections [3]. ERIC-PCR is a simple, rapid and inexpensive technique in comparison with MLST, PFGE and whole-genome sequencing (WGS) [9,10]. Additionally, it exhibits high discriminatory power and reproducibility, which favours its use for the study of the relationship between isolates of various origins, source attribution and genetic diversity. Compared with other methods, PCR-based fingerprinting methods are easier to perform and cost-effective [3].

*Campylobacter* genotyping is the final step in the characterization of these strains. It enables effective assessment of their origins and/or relationships, which is essential for adequately addressing human and animal infections [11,12]. However, data based on this technique are almost non-existent in Gabon. Consequently, this study aims to determine the genetic diversity of *C. jejuni* strains isolated from infant diarrhoeal faeces at the Omar Bongo Ondimba Regional Health Center of Makokou (CHROBOM), Gabon.

## Methods

### Bacterial strains

The biological material used in this study was obtained from the strain collection of the Medical Analysis Research Unit of the Bacteriology Laboratory housed in the CHROBOM. This material consisted of 58 *C. jejuni* strains isolated from infant diarrhoeal faeces. The samples were collected from 18 April to 18 October 2023.

### *Campylobacte*r culture

The various stool samples were cultured on modified charcoal-cefoperazone-deoxycholate agar (mCCDA) selective medium complying with ISO standards 10272–1 and 10272–2. Plates were incubated at 42 °C for 24–48 h in micro-aerophilic conditions in a 2.5 l Genbox Jar (bioMérieux, Marcy-l'Étoile, France). The colonies obtained were then screened by orientation tests: Gram staining, oxidase test and catalase test. Bacteria were identified using the Campy Identification Profile Analysis (API) gallery. The different strains were cryopreserved in brain heart infusion (BHI), supplemented with glycerol in a 70/30 ratio and stored at −40 °C. The positive control was *C. jejuni* NCTC 11168, and the negative control was *Escherichia coli*.

### Molecular identification of *Campylobacter*

For molecular confirmation of *C. jejuni* strains, primers specific for *Campylobacter*-specific thermophilic 23S rRNA were used (GenBank accession no. Z29326).

DNA extraction was performed from fresh culture, obtained following incubations on a solid medium according to the instructions of the Invitrogen Purlink DNA/RNA Extraction Kit (Thermo Fisher Scientific, USA), and the total DNA was quantified using the Qubit 4™ Fluorometer (Thermo Fisher Scientific, Wilmington, DE, USA). The DNAs of the 58 *Campylobacter* strains previously identified by API CAMPY were amplified using the conventional PCR method. The *23S rRNA* sequence used was as follows: *23* S-F (5′-TATACCGGTAAGGAGTGCTGGAG-3′), *23* S-R (5′-ATCAATTAACCTTCGAGCACCG-3′).

The amplification programme was performed as described in previous studies [13]. PCR reactions were performed using a thermal cycler (Bio-RAD, T100™, USA) in a final reaction volume of 20 µl, containing 2 µl of DNA, 10 µl of 2X master mix (Ampli Taq Golden 360, Thermo Fisher), 2 µl of sense and antisense primers (10 pmol), 1.2 µl of 1.5 mM MgCl2 and 4.8 µl of ultrapure water. The PCR conditions and amplification programmes were as follows: initial denaturation at 95 °C/6 min, followed by 30 cycles of denaturation at 95 °C/30 s, hybridisation at 59 °C/30 s, elongation at 72 °C/30 s and final elongation at 72 °C/7 min. Each reaction contained a positive control (*C. jejuni* NCTC 11168) and a negative control (*E. coli*).

The amplicons were separated by electrophoresis on a 1.5% (w/v) agarose gel prepared in a 0.5X Tris–Borate–EDTA (TBE) solution containing ethidium bromide (BET). Migration was performed at 100 V and 100 mA for 1 h and 30 min using the CONSORT EV243 (Power Supply, Belgium). These fragments were visualized using the Quantum ST4 1100/26 MX software (Vilbert Lourmat, France).

### Molecular typing of diarrhoeal *C. jejuni* strains by the ERIC-PCR method

### Amplification

DNA was extracted from colonies that were less than 48 h old from a fresh culture obtained after incubation on a solid medium. The total DNA from all the isolates was extracted according to the instructions of the *Invitrogen* Extraction Kit Purlink DNA/RNA (Thermo Fisher Scientific, USA). Genetic diversity was investigated using the ERIC-PCR method on the 58 *C. jejuni* strains. The sequence of the ERIC primers was as follows: *ERIC-F (5′* AGTAAGCTCCTGGGGATTCAC3′), *ERIC-R (5′'* AAGTAAGTGACTGGGGTGAGCG3′).

Amplification was carried out as described in a previous study [14,15]. The PCR reactions were performed using the Bio-RAD T100™ (USA) thermocycler in a final reaction volume of 20 µl, containing 2 µl of DNA, 10 µl of 2X master mix (Ampli Taq Golden 360, Thermo Fisher), 2 µl of sense and anti-sense primers (10 pmol), 1.2 µl of MgCl _2_ at 1.5 mM and 4.8 µl of ultra-pure water. The PCR conditions and amplification programmes were as follows: initial denaturation at 95 °C/5 min, followed by 30 cycles of denaturation at 94 °C/1 min, hybridization at 52 °C/1 min, elongation at 72 °C/2 min and final elongation at 72 °C/5 min.

The PCR products were separated by electrophoresis on a 1.5% (w/v) agarose gel, prepared in a 0.5X TBE solution containing 0.5 µg ml^−1^ BET. The electrophoresis was performed at a voltage of 100 V and an intensity of 100 mA for 1 h and 30 min using a CONSORT EV243 device (Power Supply, Belgium). The gel was visualized using the Quantum ST4 1100/26 MX software (Vilbert Lourmat, France).

#### Analysis of electrophoretic profile creation of clusters

The manual binomial (zero–one) method was used to analyse the models using the online data analysis service (http://insilico.ehu.es/dice_upgma/), which uses the Dice index and unweighted pair group correspondence analysis with arithmetic mean (UPGMA). It makes it possible to compare strains according to their similarities and to group the most similar individuals. The dendrogram was drawn based on the clusters. The discriminatory power of ERIC-PCR was measured by Simpson’s diversity index (*D*), which indicates the average probability that a typing system assigns a different type to two unrelated strains at random within a population [16]. A colour code has been established according to the origin of patients in each district of the town of Makokou.

A *D* value of 1.0 would indicate that the typing method was able to distinguish each member of a strain population from all other members of that population.

An index of 0.0 would indicate that all members of a population of strains were of an identical type.

An index of 0.50 would mean that there is a 50% probability that the randomly chosen strain would be indistinguishable from the first.

## Results

ERIC-PCR genetic profiles were discriminated by the number and position of amplified fragments.

**Fig. 1.**
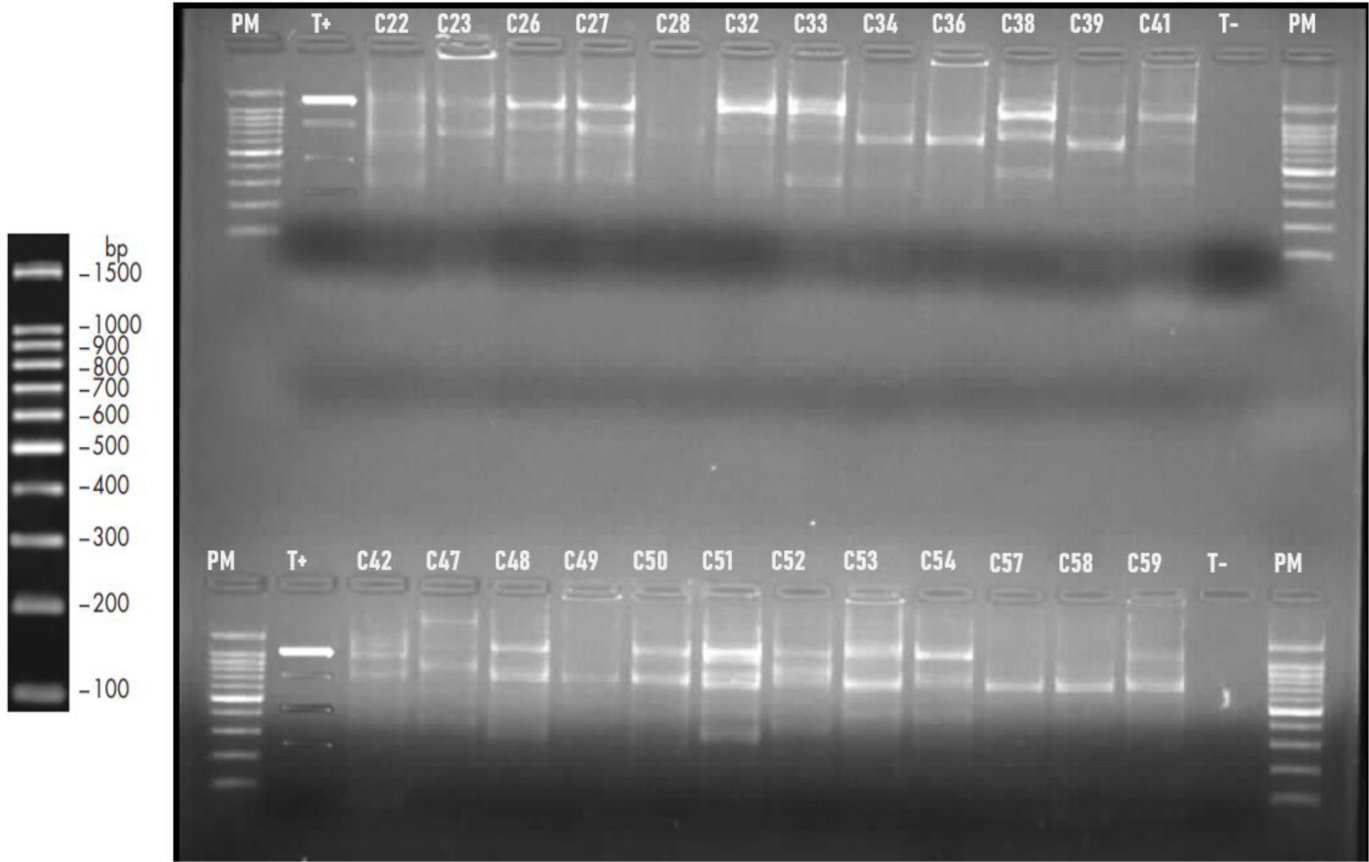
ERIC-PCR profiles of *C. jejuni* isolates from the CHROBOM (Gabon) on 1.5% electrophoretic gel. PM, molecular weight marker (1500 bp); T+, positive control; T−, negative control; C: *C. jejuni.*

Thus, the amplification of 58 strains of *C. jejuni* by ERIC-PCR recorded 89.65% (52/58) positivity (Fig. 1). A total of 52 of the 58 isolates produced genetic fingerprints of one to six bands having a size of between 250 and 1500 bp, with the exception of strains C17, C20 and C47, which had a band of more than 1500 bp. The most common bands were those of 1200, 800 and 700 bp in size.

An analysis of the genetic profile revealed that the 58 isolates were separated into 33 genotypic profiles, including 22 non-repeated profiles and 11 repeated profiles, with a diversity of 16 bands showing a strong discrimination index (*D*) of 0.8451.

**Fig. 2.**
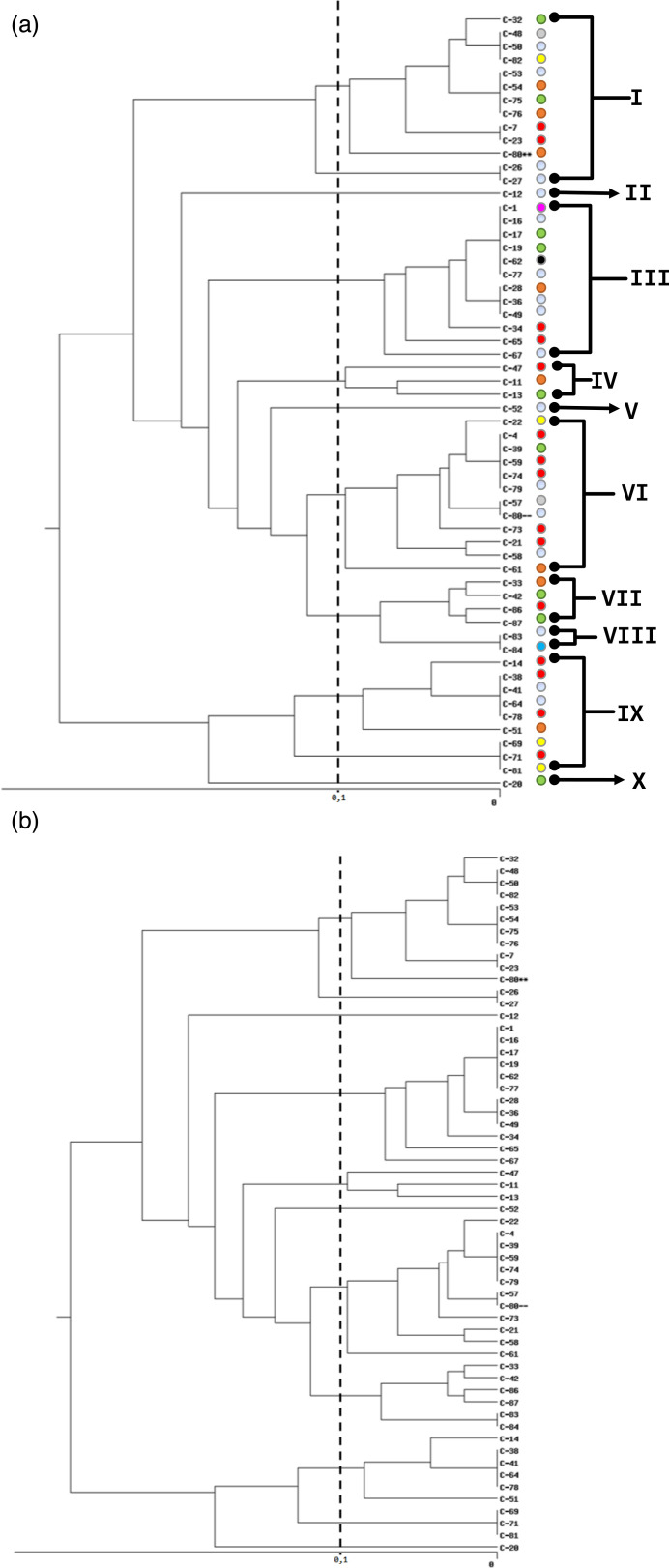
Dendrogram of *C. jejuni* isolates from the CHROBOM (Gabon) generated using UPGMA. Origin of patients according to the districts of Makokou and surrounding areas.

A dendrogram analysis showed that the 58 strains of *C. jejuni* were grouped together into ten clusters (Fig. 2 and Table 1). Cluster 1 included 13 strains, clusters 3 and 6 each had 12 strains, cluster 9 had 9 strains, clusters 4 and 7 each contained 4 strains, cluster 8 included 2 strains and clusters 2, 5 and 10 each had only 1 strain.

**Table 1.** ERIC-PCR fingerprint profiles and associated groups of *C. jejuni* isolates from the CHROBOM, Gabon

Profile	Isolate	No. of isolates	Cluster
E1	C32	1	I
E2	C48, C50, C82	3	
E3	C53, C54, C75, C75	4	
E4	C7, C23	2	
E5	C80*	1	
E6	C26, C27	2	
E7	C12	1	II
E8	C1, C16, C17, C19, C62, C77	6	III
E9	C28, C36, C49	3	
E10	C34	1	
E11	C65	1	
E12	C67	1	
E13	C47	1	IV
E14	C11	1	
E15	C13	1	
E16	C52	1	V
E17	C22	1	VI
E18	C4, C39, C59, C74, C79	5	
E19	C57, C80†	2	
E20	C73	1	
E21	C21	1	
E22	C58	1	
E23	C61	1	
E24	C33	1	VII
E25	C42	1	
E26	C86	1	
E27	C87	1	
E28	C83, C84	2	VIII
E29	C14	1	IX
E30	C38, C41, C64, C78	4	
E31	C51	1	
E32	C69, C71, C81	3	
E33	C20	1	X

“*” and “†” represent two different types of colonies on mCCDA agar obtained and Gram stains from the same patient, both of which have been identified as *Campylobacter jejuni*. They are characterized by: for “*”, a flat colony with a grey, metallic serrated edge, Gram-negative coccoid. For “†”, a grayish colony with a metallic sheen, flat and moist, curved bacillary Gram negatives.

Furthermore, the distribution of different profiles and clusters of circulating *Campylobacter* strains within neighbourhoods is heterogeneous.

## Discussion

The study aimed to determine the genetic diversity of *C. jejuni* strains isolated from infant diarrhoeal faeces at the CHROBOM.

After performing ERIC-PCR fingerprinting, the result showed 16 bands ranging from 250 to over 1500 bp clustered using the UPGMA dendrogram. This finding is different from that of Staji *et al.* [5], who reported 25 bands of *C. jejuni* ranging from 400 to 735 bp from 60 isolates of chicken shreds and faeces from Semnān City in Iran. This difference could be explained not only by the difference in the choice of the study specimen from which the samples were collected but also by the use of ERIC whose optimized primers lead to better typicity and even higher discriminatory power [17]. Indeed, the study by Alsultan and Elhadi [17] carried out on *E. coli* suggested a clear added value for the use of a single ERIC1 or ERIC2 primer for the determination of diversity via ERIC-PCR in Gram-negatives. Moreover, the number of different bands observed suggested the presence of ample genetic diversity in the *C. jejuni,* as reported by Igwaran and Ifeanyi in [3], who used the ERIC-PCR method to investigate strains of *C. coli* and *C. jejuni* in the district of Chris Hani and the municipalities of the districts of Amathole in South Africa.

In the present study, the high positivity rate of 89.65% of *C. jejuni* strains, combined with the 33 profiles (22 non-repetitive and 11 repetitive) obtained for the isolates examined, indicates the genetic heterogeneity of the strains studied. This high level of heterogeneity in *C. jejuni* is probably linked to two factors: the great plasticity of the genome of the *Campylobacter* genus, making it relatively unstable [10], and the numerous changes within the genome during colonization cycles or infection, leading to natural changes, genomic rearrangements and chromosomal point mutations, resulting in new genotypes [3,8]. It could also be the result of the epidemiology of several genetically different clones, or a clone that has evolved over time [18].

The presence of different clusters in the different neighbourhoods suggests the clonal dissemination of *Campylobacter* strains. This spread could be conveyed by waterways, the presence of domestic animals and/or non-regulated livestock and faecal carriage or the lack of hygiene by the populations of this city [19,20].

The 58 *C. jejuni* strains were separated into ten clusters with a discriminatory power *D*=0.8451 and a heterogeneous distribution of the strains in Makokou’s neighbourhoods. Indeed, the discriminative power obtained in this study is very close to that of Ramees *et al.* [21], who reported *D*=0.83 and 0.82, respectively, for *C. jejuni* and *C. coli,* suggesting ERIC-PCR’s strong capacity for discriminating strains of the same species compared with other very expensive techniques [22]. Genetic DNA fingerprinting tests, such as ERIC-PCR, can facilitate the analysis of genomic similarities between different samples and their categorisation into clusters [23]. Staji *et al.* [5] effectively emphasized a better detection of genotypic profiles with the ERIC-PCR method compared with RAPD-PCR in their 2018 study of *C. jejuni* isolates from poultry from Semnān City in Iran. Thus, the heterogeneous distribution of strains grouped into ten clusters circulating within the city’s neighbourhoods would be a fairly accurate representation of the clonal circulation of *C. jejuni* within the city of Makokou. However, further investigations would help provide more data on the epidemiology of *Campylobacter* in the study area because high discriminatory power from the ERIC-PCR method alone does not always correspond to a precise representation of epidemiological links [11].

## Conclusion

In summary, these results not only just report the first cases of isolation and genetic characterization of *C. jejuni* in the town of Makokou. These strains are genetically diverse and heterogeneously distributed, suggesting an epidemic of diarrhoea in the town.

It is indeed crucial to highlight the genetic diversity observed and its potential implication in a diarrhoea epidemic in the region. The use of *23S rRNA* to confirm biochemical identification is an important step. However, as you mention, the integration of other molecular tools, such as *16S rRNA* and the *hipO* and *MapA* genes, could enrich the results and provide a more complete understanding of the situation. In addition, it would have been desirable to establish a link between the resistance profiles of these strains and the phylogenetic profiles obtained by ERIC-PCR. In addition, exploring aquatic and food systems to identify sources of contamination would be an excellent suggestion. This could help not only to understand the current epidemic but also to prevent future infections. Indeed, the need for further studies on the presence of *Campylobacter* in food and drinking water is crucial to improving awareness systems and diagnostic techniques in public health establishments in this region of Gabon. Finally, the study of genetic heterogeneity or diversity would have been more thorough and specific through the use of techniques such as MLST and WGS in order to improve and better understand the circulation and origin of strains.

## Abbreviations

API: identification profile analysis
BET: ethidium bromide
CHROBOM: Omar Bongo Ondimba Regional Health Center of Makokou
ERIC-PCR: enterobacterial repetitive intergenic consensus PCR
mCCDA: modified charcoal-cefoperazone-deoxycholate agar
TBE: Tris–Borate–EDTA
UPGMA: unweighted pair group correspondence analysis with arithmetic mean
WGS: whole-genome sequencing
